# Hot carrier multiplication on graphene/TiO_2_ Schottky nanodiodes

**DOI:** 10.1038/srep27549

**Published:** 2016-06-08

**Authors:** Young Keun Lee, Hongkyw Choi, Hyunsoo Lee, Changhwan Lee, Jin Sik Choi, Choon-Gi Choi, Euyheon Hwang, Jeong Young Park

**Affiliations:** 1 Center for Nanomaterials and Chemical Reactions, Institute for Basic Science (IBS), Daejeon 305-701, Korea; 2 Graduate School of EEWS, Korea Advanced Institute of Science and Technology (KAIST), Daejeon 305-701, Korea; 3 Creative Research Center for Graphene Electronics, Electronics and Telecommunications Research Institute (ETRI), Gajeongno, Yuseong-gu, Daejeon, 305-700, Republic of Korea; 4 SKKU Advanced Institute of Nanotechnology, Sungkyunkwan University, Suwon 440-746, Republic of Korea

## Abstract

Carrier multiplication (i.e. generation of multiple electron–hole pairs from a single high-energy electron, CM) in graphene has been extensively studied both theoretically and experimentally, but direct application of hot carrier multiplication in graphene has not been reported. Here, taking advantage of efficient CM in graphene, we fabricated graphene/TiO_2_ Schottky nanodiodes and found CM-driven enhancement of quantum efficiency. The unusual photocurrent behavior was observed and directly compared with Fowler’s law for photoemission on metals. The Fowler’s law exponent for the graphene-based nanodiode is almost twice that of a thin gold film based diode; the graphene-based nanodiode also has a weak dependence on light intensity—both are significant evidence for CM in graphene. Furthermore, doping in graphene significantly modifies the quantum efficiency by changing the Schottky barrier. The CM phenomenon observed on the graphene/TiO_2_ nanodiodes can lead to intriguing applications of viable graphene-based light harvesting.

When photons impact photon absorbers, such as a semiconductor and a metal, electron–hole pairs and hot carriers are produced through energy conversion from the photon energy. The conversion efficiency of photon energy harvesting has been enhanced in various ways, such as tandem structures, bandgap engineering, or plasmonics^1^,^2^,^3^,^4^,^5^. These days, semiconductor-based energy harvesting has overcome the maximum power efficiency of solar energy conversion, which is known as the Shockley–Queisser limit^6^. One of the factors behind the Shockley–Queisser limit is the difference in energy between the incident photon energy and the minimum energy that can produce an electron–hole pair, which is finally dissipated as heat. Nozik introduced quantum dot solar cells that simultaneously operate via hot carriers and a collection of secondary electrons generated from hot carriers before thermalization, proposing the optimistic possibility for increasing the theoretical maximum efficiency^7^. A single high-energy photon is absorbed on a photon absorber and, as a result, more than two electron–hole pairs are generated before the excess energy is lost as heat (i.e. carrier multiplication (CM))^8^,^9^,^10^,^11^. Schaller *et al.* investigated CM in PbSe nanocrystals (NCs) and the optimal bandgap in highly efficient PbSe-NC-based solar cells^12^. The process of CM occurs not only in semiconductor NCs, but also in the bulk semiconductor. Pijpers *et al.* demonstrated that the efficiency of CM in the bulk semiconductor was more efficient than in semiconductor NCs, which results from the reduced density of states in NCs compared with bulk materials^13^.

Recently, CM in graphene has attracted a lot of theoretical and experimental interest because of its sensitive photon response^14^. Graphene is emerging as a promising material for optoelectronics because of the unique optical and electronic properties originating from its linear and gapless band structure as well as its flexibility and easy transfer to various substrates. After generating a photoexcited carrier in graphene, energy relaxation from the photoexcited carrier generally undergoes two types of scattering processes: phonon emission (i.e. carrier–phonon interaction) and carrier–carrier interaction, which can contribute to the thermalization of carriers^15^,^16^. In the phonon emission process, the energy of the excited carrier is transferred as heat. On the other hand, in the carrier–carrier interaction process, the photoexcited carrier relaxes its energy by producing secondary electrons below the chemical potential. The relaxation process via electron–electron interaction is much shorter than the electron–phonon interaction in graphene^17^. Using optical pump–terahertz probe measurements, Tielrooij *et al.* demonstrated that the carrier–carrier interaction is the predominant process in graphene during energy relaxation of energetic photoexcited electrons at femtosecond scale^11^. CM in graphene originates from very short carrier–carrier interaction scattering, which is also known as impact ionization^18^. Thus, optically excited hot electrons scatter with other electrons below the Fermi level through the electron–electron interaction before thermalization by emitting phonons, which occurs at picosecond scale. The final number of excited electron–hole pairs depends on the energy of the primary photoexcited electron and the probability of the impact ionization process. In graphene, it is noted that impact ionization is an efficient pathway for energy dissipation, compared with semiconductors that are limited because of bandgap and energy dispersion issues^8^. For a better understanding of favorable impact ionization, a theoretical approach to relaxation dynamics in graphene under Landau quantization was reported by Wendler F. *et al.*^19^ to solve the challenge of carrier extraction in a gapless structure by finding a situation (e.g. pump fluence, temperature, magnetic field, Landau level broadening) for efficient impact excitation.

In this paper, we fabricated graphene/TiO_2_ Schottky nanodiodes to utilize CM in graphene as an energy harvesting route by which hot carriers are produced and detected through the Schottky barrier. Hot electrons are energetic electrons that are not at thermal equilibrium with the lattice. The energetic electrons lose their energy through inelastic scattering processes to generate secondary electrons within the mean free path of the electrons^20^. Previously, we investigated energy transfer to hot electrons from photons in Au/TiO_2_ Schottky nanodiodes and observed that hot electron generation can be enhanced by localized surface plasmons, which increases the quantum efficiency of the device^4^. It is well known that surface plasmons of metals give rise to an enhanced quantum yield of a device in visual light because the energies of the metal surface plasmons are in that range. In a graphene-based nanodiode, however, we cannot expect enhancement of the quantum yield from plasmons because graphene plasmon energy is lower than that of the Schottky barrier (i.e. below infrared). Thus, the hot electrons produced by surface plasmons cannot escape the graphene. Even though the low-energy surface plasmons of graphene do not produce hot electrons, we can still expect an enhanced quantum yield from graphene-based nanodiodes via the CM process because the CM mechanism produces many hot electrons from a single photon. In this paper, we investigate CM in graphene and by utilizing that mechanism, we show how CM can enhance quantum yield in the graphene/TiO_2_ nanodiode.

## Results and Discussion

**Figure 1:**
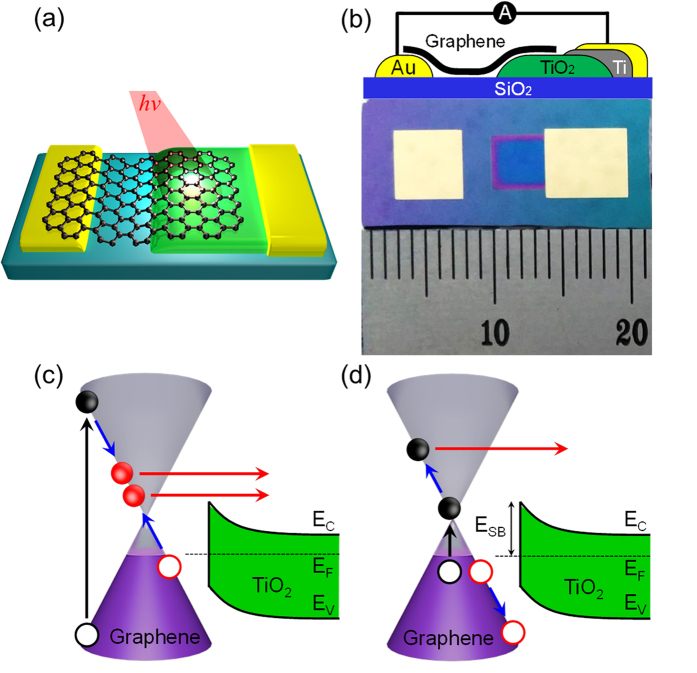
Scheme and energy band diagram of the graphene/TiO_2_ Schottky nanodiode. (**a**) Scheme and (**b**) cross–sectional view (photograph) of the graphene/TiO_2_ Schottky nanodiode. Energy band diagrams for hot CM by (**c**) impact ionization and (**d**) inverse Auger process from photon absorption on the graphene/TiO_2_ Schottky nanodiode. Here, the filled (empty) dots indicate electrons (holes).

For fabrication of the graphene/TiO_2_ Schottky nanodiode, chemical vapor deposition (CVD)–grown graphene was transferred to an n–type TiO_2_ layer. The number of graphene layers on the TiO_2_ was controlled: single (SLG), double (DLG), and triple (TLG) layers. Figure 1a shows a scheme of the graphene/TiO_2_ nanodiode, which consists of two electrodes (yellow), a TiO_2_ layer (green), and a graphene layer (black). The actual size and cross–sectional view of the nanodiode are shown in Fig. 1b. The contact between the graphene and TiO_2_ layers builds the Schottky barrier, which can play the role of hot electron collector. The Ohmic contact is formed at the interface between the TiO_2_ and Ti (Fig. 1b). Figure 1c,d represents multiple hot electron generation in the graphene/TiO_2_ nanodiode by impact ionization and the Auger process, respectively. The Fermi level of pristine graphene could be located below the Dirac point, indicating p–type doping as a result of oxygen and moisture adsorption under ambient conditions^21^. The Schottky barrier was formed at the interface between the graphene and TiO_2_ layers. When the graphene layer absorbs photons, hot electrons are produced by direct transition from the valence band to the conduction band in the graphene. The primary photoexcited electrons (black dots) lose their energy through impact ionization before being thermalized and producing phonons. The loss of excess energy from the hot electrons leads to multiple secondary electrons (red dots), which may have enough energy to overcome the Schottky barrier, as shown in Fig. 1c. In addition to CM by impact ionization, the Auger process may also contribute to the photocurrent (Fig. 1d). The photoexcited electrons with energies less than the Schottky barrier may scatter with electrons in the valence band and, as a result, the energy of the excited electrons is larger than the barrier and these electrons contribute to the photocurrent. Although both of these processes generate hot carriers, the dominant pathway for hot carrier generation is impact ionization. Additionally, the inverse Auger process may contribute to CM. In the inverse Auger process, low-energy electrons usually gain energy by scattering with holes. However, in our experiment, the electrons are photo-excited with very high energy, and therefore the decay of hot electrons through electron–electron interactions may prevail over the inverse Auger process. In addition to hot electrons from graphene, photons with energy higher than the bandgap of TiO_2_ can be absorbed in the TiO_2_ layer (band-to-band excitation). Thus, in this case, the total photocurrent is attributed to the excited electrons (i.e. hot electrons on the graphene or the metal film) from both graphene and TiO_2_.

**Figure 2:**
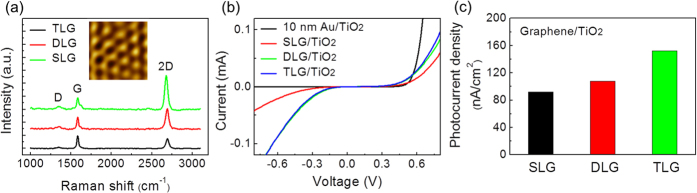
Raman spectra and electrical measurements. (**a**) Change in Raman spectra of the graphene (Inset: atomic stick–slip image of SLG, 2 × 2 nm.), (**b**) *I*–*V* curves, (**c**) photocurrent density measured on the graphene/TiO_2_ diodes as a function of the number of graphene layers (SLG: single–layer graphene, DLG: double–layer graphene, TLG: triple–layer graphene).

Figure 2 shows the Raman and electrical characteristics of the graphene/TiO_2_ Schottky nanodiode. Figure 2a shows Raman spectra using a 514 nm–wavelength laser (2.4 eV) recorded on single-, double-, and triple-layer graphene transferred onto TiO_2_; an atomic stick–slip image of SLG on the nanodiode is shown in the inset. The ratio of the intensities of the 2D and G peaks decreases as the number of graphene layers increases. The tendency of the intensity ratio of the 2D to G peaks shows an obvious thickness dependence (i.e. 3.1 (SLG) > 1.7 (DLG) > 0.79 (TLG)) and the 2D peak becomes broad and blue shifted, which are typical behaviors of Raman spectra as the number of layers increases^22^,^23^,^24^. To confirm the formation of the Schottky junction, current–voltage curves were measured on the graphene/TiO_2_ diodes (Fig. 2b). From the current–voltage curves, the electrical factors of the nanodiodes (e.g. the Schottky barrier height, ideality factor, and series resistance) were obtained by fitting the current–voltage curves to the thermionic emission equation (Fig. S1). Alternatively, we determined the Schottky barrier heights of the nanodiodes from the temperature dependence of the reverse saturation current (Fig. S2), which are consistent with the values from fitting to the thermionic emission equation. The Schottky barrier height is necessary to fit the incident photon-to-current conversion efficiency (IPCE) to Fowler’s law to detect multiple hot electron generation^25^,^26^,^27^,^28^. Figure 2c shows the photocurrent density measured under illumination by a tungsten–halogen lamp (9 mW/cm^2^). The photocurrent density increased as the number of layers of graphene increased because of enhanced absorption of incident light on the multilayer graphene.

To detect the generation of hot electrons on the Au and graphene layers, the energy conversion efficiency was measured as a function of the photon energy. The IPCE was fitted to Fowler’s law to confirm the generation of hot electrons from the excitation of valence electrons in the graphene and Au film by incident photons. As the photon energy increased above the Schottky barrier energy, there was an increase in the fraction of valence electrons with energy below the Fermi level that could be excited and travel over the Schottky barrier. The conversion efficiency as a function of the photon energy is given by


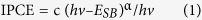



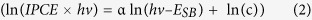


where c is a constant, *hv* is the photon energy (*v* being the photon frequency), and *E*_*SB*_ is the Schottky barrier height. For most metals, α = 2; other exponents apply to semiconductors^26^,^29^.

**Figure 3:**
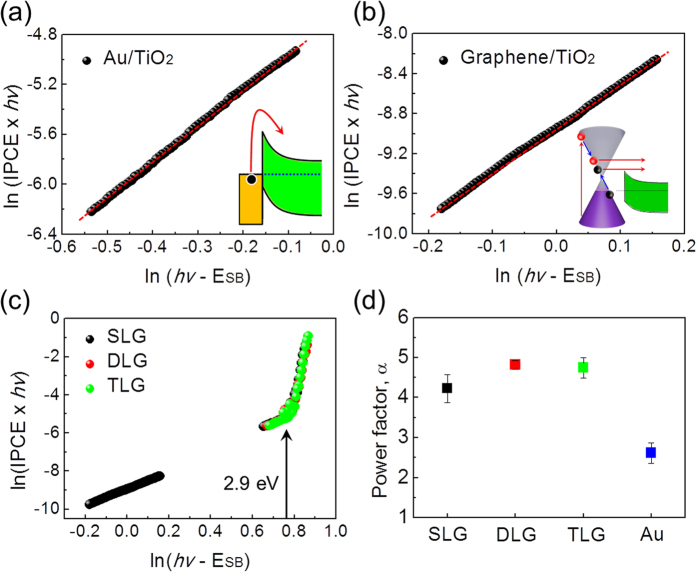
Plots of ln(*IPCE* × h*v*) as a function of ln(*hv* − *E*_*SB*_) and power factor α. Plots of ln(*IPCE* × *hv*) as a function of ln(*hv* − *E*_*SB*_) in (**a**) a 10 nm–thick Au film/TiO_2_ diode and (**b**) a graphene/TiO_2_ diode. The slope in the visible wavelength represents the exponent α in Fowler’s law (i.e. the slope in ln(*IPCE* × *hv*) vs. ln(*hv* − *E*_*SB*_)). (**c**) Plots of ln(*IPCE* *×* *hv*) as a function of ln(*hv* − *E*_*SB*_) in the graphene/TiO_2_ diodes exhibiting band-to-band excitation behavior (above 2.9 eV) in the TiO_2_ layer. (**d**) The exponent α in Fowler’s law obtained from (**a,b**).

Figure 3a,b shows the IPCE as a function of photon energy (i.e. ln(*IPCE* × *hv*) vs. ln(*hv* − *E*_*SB*_)) that is modified from Fowler’s law to determine the exponent, α. As shown in Fig. 3c, (*IPCE* × *hv*) can be characterized by two distinct behaviors (i.e. two different slopes), which are separated by the bandgap energy of TiO_2_ (*E*_*g*_). The position of the black vertical arrow corresponds to the bandgap (*E*_*g*_, 2.9 eV) of TiO_2_ as the boundary between the two distinct behaviors (i.e. photoemission from thin gold/graphene and from direct interband excitation in the TiO_2_ layer). It is obvious that band–to–band excitation of TiO_2_ is dominant for generating photocurrent above the photon energy (i.e. higher than ~2.9 eV), which is close to the band gap of TiO_2_. On the other hand, for photon energy below ~2.8 eV, the IPCE as a function of the photon energy has a different slope because the generation of hot electrons is the main pathway for producing photocurrent.

In the energy range lower than the bandgap, (*IPCE* × *hv*) increases as a power law in terms of (*hv* − *E*_*SB*_)^α^. We found that α is 2.6 ± 0.26 and 4.2 ± 0.35 for gold and SLG, respectively, for *hv* < *E*_*g*_ (Fig. 3d). The main contribution to the photocurrent in this region arises from hot electrons from the photon–absorbing layers (e.g. graphene and gold) because direct excitation in the TiO_2_ is not allowed. When the photoexcited electrons on the metal surface escape the interface, the collected photocurrent can be expressed by Fowler’s law (i.e. (*IPCE* × *hv*) ∝ (*hv* − *E*_*SB*_)^α^ where α = 2)^26^,^29^. We found that in the Au/TiO_2_ nanodiode, α = 2.6 ± 0.26 is exhibited for *hv* < *E*_*g*_, indicating that the photocurrent was induced from photoexcited electrons in Au. However, in the graphene/TiO_2_ nanodiode, we observed α = 4.2 ± 0.35, which deviates from Fowler’s law. This deviation implies that additional photocurrent mechanisms can exist in the graphene/TiO_2_ nanodiode. We also found that the exponent α in Fowler’s law with gapless and linear energy dispersion of carriers like graphene is 3, and the exponent cannot be larger than 3 because the exponent for this case is largely determined by the density of states (DOS) of the system. With parabolic dispersion (i.e. with square-root energy-dependent DOS), we have the usual exponent value of 2^30^,^31^. To get an exponent larger than 4, we must consider the multiplication of carriers with energy higher than the Schottky barrier. Thus, the high value of the exponent in our quantum yield experiment can be understood in terms of CM instead of the role of DOS in the emitter.

To quantitatively identify this anomalous behavior, we considered the CM process of hot carriers in graphene. By incorporating the CM process of photoexcited electrons, we determined that (*IPCE* × *hv*) ∝ (*hv* − *E*_*SB*_)^4^. To find the photocurrent, we calculated the total number of electrons with energies normal to the 2D plane of graphene and higher than the Schottky barrier *E*_*SB*_. For hot electrons to travel over the surface barrier, the energy component normal to the 2D plane of graphene must be greater than the Schottky barrier. The escape probability is assumed to be unity if this condition is satisfied regardless of the in-plane electron energy in the graphene layer. It is intuitive to consider only carrier generation and escape steps for carrier transport normal to the 2D plane of graphene. By considering the CM process for hot electrons with a normal component of energy greater than the Schottky barrier, we find the total number of hot electrons *n* after CM,


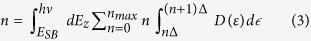


where *n*_*max*_ = int*(hv*−*E*_*z*_), *ε* is the in-plane energy, *D*(*ε*) is the density of state of graphene, and ∆ is the characteristic average energy loss per multiplication step, which is on the order of the Fermi energy^18^. Near the bandgap energy of TiO_2_, we found that *n* ∝ (*hv* − *E*_*SB*_)^4^. Thus, CM in graphene can be detected by measuring the exponent α, which can be determined by the slope of the log–log plot of (*IPCE* *×* *hv*) as a function of (*hv* − *E*_*SB*_). The graphene/TiO_2_ nanodiode exhibits α = 4.2 ± 0.35, which represents the effect of CM in graphene on hot electron generation. Figure 3d shows the exponent α for different layers of graphene and for a thin gold layer. The measured exponents are slightly larger than the calculated value. The small deviation can be understood from the higher-order contributions of the above equation to the photocurrent. We note that the exponent α = 4 does not depend on the number of graphene layers. The efficiency of CM is determined by the relaxation time of the carriers through carrier–carrier scattering. If carrier relaxation is dominated by the carrier–carrier scattering time, the hot carriers decay through CM. It is known that for the linear energy dispersing region (i.e. graphene, high-energy region in DLG/TLG), the relaxation time by carrier–carrier scattering is always shorter than by thermalization (i.e. electron–phonon scattering). Therefore, the CM in DLG/TLG is equivalent to CM in monolayer graphene for hot carriers.

The transport possibility for hot electrons generated by the CM process is directly related to the Schottky barrier height. It is expected that as the Schottky barrier height decreases, hot electron transport becomes more efficient because energetic electrons can more easily overcome the lower Schottky barrier. Therefore, lowering the Schottky barrier height can induce an increase in photocurrent, improving the quantum yield in a graphene/semiconductor nanodiode. Also, the Schottky barrier height in the photodiode determines the threshold energy above which the photocurrent is observed by photoemission. CM characteristics can be manipulated by doping (or chemical potential) of the graphene. Recently, a change in the CM factor dependent on doping was reported by Johannsen *et al.*^32^ The measured CM factor in electron-doped (i.e. n-type) graphene was roughly three times larger than that of hole-doped (i.e. p-type) graphene. Upon light illumination, the main mechanism for carrier excitation is interband transition. Primary excitation by interband transition can produce secondary electrons through impact ionization. The efficiency of charge transport of the secondary electrons over the Schottky barrier can be affected by the change in Fermi level. In the graphene/TiO_2_ Schottky nanodiode, doping modifies the Schottky barrier height. As the Schottky barrier increases (i.e. for hole-doped graphene), the number of hot carriers with energy high enough to overcome the Schottky barrier decreases and therefore the diode has low efficiency for internal photoemission. Even though the quantum efficiency depends on the Schottky barrier height (or doping), we show that the exponent α in Fowler’s equation is independent of the barrier height. Thus, we also confirmed the effect of CM on the graphene/TiO_2_ nanodiode by investigating the doping effects.

**Figure 4:**
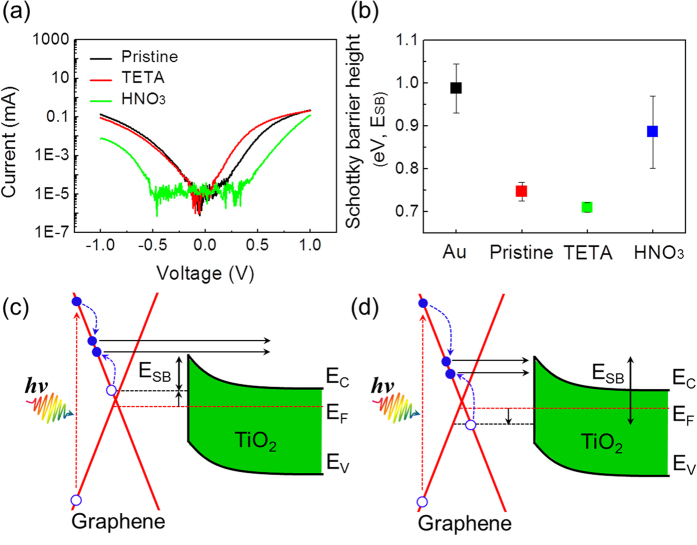
Electrical measurements after doping and possible modifications of the energy band diagram. (**a**) Current–voltage curves after doping. (**b**) Change in Schottky barrier height dependent on doping. Illustrations of internal photoemission after impact ionization dependent on modification of the Schottky barrier in (**c**) the TETA-doped graphene/TiO_2_ and (**d**) the HNO_3_-doped graphene/TiO_2_ Schottky diode.

To dope the graphene, triethylene tetramine (TETA) and nitric acid (HNO_3_) were used for n- and p-type doping, respectively^33^,^34^,^35^. Details about the doping of graphene and characteristics of the doped graphene are described in the Methods and in Figs S3 and S4. The significant change in graphene caused by doping is the Schottky barrier height as a function of the type of doping. The n–type (p–type) doping can decrease (increase) Schottky barrier height because of an upshift (downshift) of the Fermi level. After doping the graphene, current–voltage curves were measured on the doped graphene/TiO_2_ Schottky nanodiode to confirm the change in electrical properties (Fig. 4a) depending on doping, indicating the effects of doping on the electrical properties (e.g. Schottky barrier height and series resistance (Fig. S5)). The Schottky barrier height was calculated by fitting the current–voltage curves to the thermionic emission equation, as shown in Fig. 4b. For the graphene/TiO_2_ Schottky contact, *E*_*SB*_ is expected to be *E*_*SB*_ = Φ_G_ − χ, where Φ_G_ is the work function of the graphene and χ is the electron affinity of the semiconductor. It is noted that the electron affinity of TiO_2_ is about 4.0 eV^36^,^37^. Therefore, it is expected that the work function of the graphene is about 4.7 eV, which is in good agreement with the literature^38^,^39^. The Schottky barrier in the graphene/TiO_2_ diodes could be affected by the p-doping effect at ambient conditions. As the p-doping effect is exhibited on the graphene/TiO_2_ diodes, the Schottky barrier height increases because the Fermi level of the graphene decreases from the Dirac point, and the work function of the graphene thus increases. In n-type (p-type) doping conditions, the Schottky barrier decreased to 0.71 ± 0.01 eV (increased to 0.89 ± 0.08 eV), compared with the pristine graphene/TiO_2_ Schottky nanodiode at 0.75 ± 0.02 eV. The manipulation of the Schottky barrier height *E*_*SB*_ in the graphene/TiO_2_ Schottky diode is illustrated as a function of doping in Fig. 4c,d, thus implying a change of probability for hot carrier transport over the *E*_*SB*_ after effective impact ionization. Upon the decrease of the Schottky barrier in the TETA-doped graphene/TiO_2_ Schottky diode, the probability of internal photoemission after impact ionization can be enhanced, as indicated in Fig. 4c. On the other hand, in the HNO_3_-doped graphene/TiO_2_ Schottky diode, the increase in the Schottky barrier hinders internal photoemission because more energy is needed for the hot electrons to overcome the higher Schottky barrier, as depicted in Fig. 4d.

**Figure 5:**
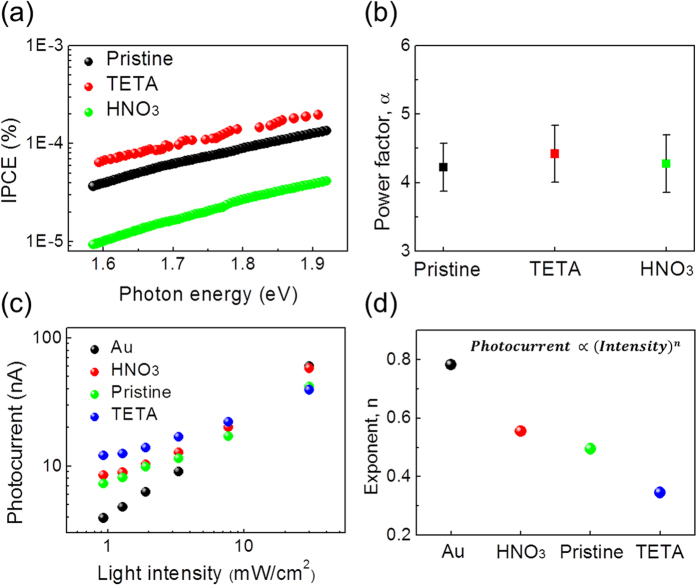
IPCE, power factor α, and dependence of photocurrent on light intensity on the graphene/TiO_2_ Schottky nanodiodes. (**a**) IPCE measured on the graphene/TiO_2_ Schottky nanodiodes after doping. (**b**) Power factor α obtained by fitting the IPCE to Fowler’s law as a function of doping. (**c**) Photocurrent measured on the Au/TiO_2_ and SLG/TiO_2_ Schottky nanodiodes as a function of light intensity. The photocurrent was measured as a function of the incident light intensity. The photocurrent of the SLG/TiO_2_ shows a very weak intensity dependence; at the low light intensity limit (<8 mW/cm^2^), the photocurrent of SLG/TiO_2_ is larger than that of Au/TiO_2_, resulting from efficient hot electron generation through CM at weak light intensity. The TETA-doped graphene/TiO_2_ shows the lowest intensity dependence of photocurrent, exhibiting an enhanced effect of CM by an increase in the effective impact ionization and internal photoemission from a decrease in the Schottky barrier. (**d**) The values of exponent *n* representing the relationship between photocurrent and light intensity.

Figure 5a shows IPCE measured on graphene/TiO_2_ Schottky diodes after TETA and HNO_3_ doping. Based on the IPCE of the graphene/TiO_2_ Schottky diodes, the power factor α after doping was obtained by fitting the IPCE to a modified Fowler’s law, as shown in Fig. S6. The power factors were all about 4.3, regardless of doping (Fig. 5b). This is precisely what we expected from the theoretical analysis based on CM. Doping changes the Fermi energy of the graphene and the number of hot electrons. However, the exponent α is entirely determined by the density of states. In the equation for the number of hot electrons given on page 6, ∆ and *n*_*max*_ changed after doping, but the density of states remains the same. Therefore, the number of hot electrons (i.e. equivalently, the photocurrent) depends on the doping because of the change in ∆ and *n*_*max*_, but we still have the same energy dependence from the equation (i.e. the same exponent α) because the density of states does not change. We note that additional doping may increase the efficiency of Auger recombination; therefore the IPCE does depend on the doping.

When the main mechanism for photocurrent is governed by CM, the exponent in Fowler’s law is independent of barrier height, even though the quantum efficiency is determined by the barrier height. We also confirm the CM effects in the graphene/TiO_2_ nanodiode by observing the intensity-dependent photocurrent of the incident light. In Fig. 5c, we show the photocurrent as a function of the intensity of incident light for various samples with different doping. The slope of the photocurrent as a function of light intensity represents the intensity dependence of the photocurrent. The measured photocurrent decreased as the light intensity decreased because the number of excited electrons decreased, in general, as the intensity of the incident light decreased due to the weak-intensity light having fewer photons.

In detail, the intensity dependence of the photocurrent originates from the nature of hot electron generation. The photocurrent from the visible wavelengths is mainly attributed to the hot electrons generated in the graphene and Au film because the bandgap of the TiO_2_ is around 2.9 eV. Therefore, the behavior of the photocurrent as a function of light intensity can represent the origin of the hot electrons, which is associated with the graphene (CM) and the Au film. To distinguish the effect of the graphene (CM) and the Au film, the relationship between photocurrent and light intensity is investigated by


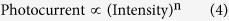


The exponent *n* can provide information about each effect of the graphene (CM) and the Au film. The intensity dependence of the photocurrent in the Au/TiO_2_ nanodiode is much stronger than in the graphene/TiO_2_ nanodiode, as shown in Fig. 5d. In addition, the photocurrent in the TETA-doped graphene/TiO_2_ exhibits very low intensity dependence, whereas the HNO_3_-doped graphene/TiO_2_ shows a higher dependence on intensity. The effect of CM is strongly observed at low light intensity^9^. A possible explanation for this unique response to light intensity of the photocurrent in doped graphene is based on two mechanisms: (1) highly efficient hot carrier transport (internal photoemission) due to the lower Schottky barrier and (2) an increase in the effective impact ionization due to an upshift of the Fermi level. As a consequence of doping, the tendency for photocurrent at the highest light intensity reverses at the lowest light intensity because more-effective CM occurs with lower light intensity, compared with the Au/TiO_2_ nanodiode. Our findings indicate that it is possible to detect hot carriers as photocurrent generated by CM in graphene using a Schottky barrier (internal photoemission), suggesting that graphene-based photodiodes can work very effectively under a dim light source. A recent report carried out by Kadi F. *et al.* has theoretically and experimentally dealt with the doping behavior for efficient CM in graphene^40^. Subsequent experiments by controlling the doping concentration in the graphene can be beneficial for further understanding of the CM detected directly using a graphene-based Schottky diode and, therefore, can shed light on the possibility for graphene-based device applications.

In conclusion, we fabricated graphene/TiO_2_ Schottky nanodiodes and observed CM-induced hot electron generation as photocurrent. The generation of hot electrons by CM in graphene can be observed by directly comparing Fowler’s law for photoemission and the power law behavior of CM. The CM mechanism in the nanodiode is also confirmed from the induced photocurrent in terms of the Schottky barrier height (or doping level of graphene) and the intensity of the incident light. As expected, the exponent in Fowler’s law is independent of barrier height, even though quantum efficiency is strongly related to barrier height. The weak dependence of photocurrent on the intensity of light also indicates strong CM effects in graphene. Thus, based on the light-intensity dependence of the photocurrent, we find that TETA- (HNO_3_−) doped graphene exhibited strong (weak) CM. We observed that in TETA-doped graphene/TiO_2_, the photocurrent decreased very slowly as the light intensity decreased, which is different from the behavior of metal-based nanodiodes where the diode current shows a very strong dependence on light intensity. The observed behavior of CM in graphene suggests feasible applications, such as graphene-based ultrasensitive photodetectors, through the detection of hot electrons amplified by CM.

## Methods

### Fabrication of nanodiodes

To detect hot electron flows, we fabricated Au/TiO_2_ and graphene/TiO_2_ Schottky diodes. The details of the device fabrication are described elsewhere^41^,^42^,^43^. In short, an insulating p-type silicon wafer covered by 500 nm SiO_2_ is prepared to electrically insulate the silicon wafer and the titanium oxide layer on the Schottky diodes. Then, a 4 × 6 mm, 150 nm-thick film of titanium is deposited onto the silicon oxide through an aluminum shadow mask using electron beam evaporation. To make titanium oxide, the titanium-deposited wafer is then annealed in air at 470 °C for 2 hours, which oxidizes the Ti to TiO_2_ and produces oxygen vacancies in the TiO_2_ film, leading to the formation of n-type TiO_2_. The next step is deposition of a 50 nm film of titanium and then a 150 nm film of gold through a second mask using electron beam evaporation, which constitutes the nanodiode’s two Ohmic electrodes. Finally, for fabrication of the Au/TiO_2_ nanodiode, a thin gold film (10 ± 2 nm thick) is deposited through a third mask by electron beam evaporation for formation of the Schottky contact between the thin Au film and TiO_2_. For fabrication of the graphene/TiO_2_ nanodiode, CVD–grown graphene is transferred onto the TiO_2_ layer. The SLG is grown by CVD on a 25 um-thick Cu foil (99.999% copper foil, Alfa Aesar). The Cu foil is loaded in a quartz tube furnace, and then heated to 1000 °C under vacuum conditions (8.9 × 10^−2^ Torr) with a constant flow of H_2_ (10 sccm) for pre-annealing. SLG is grown by flowing gas mixtures of CH_4_ (30 sccm) and H_2_ (10 sccm) under vacuum conditions (3.9 × 10^−1^ Torr) for 20 minutes. After graphene growth, a rapid cooling step follows under Ar (100 sccm) gas. To transfer the graphene to the TiO_2_ substrate, the graphene on Cu foil is spin-coated with a PMMA (poly(methyl methacrylate)) supporting layer. After the back of the graphene on Cu foil is etched by O_2_ plasma treatment (30 W, 30 s), the Cu foil is etched in 0.1 M ammonium persulfate solution. The rinsed PMMA/graphene films are transferred onto the TiO_2_ substrate and annealed at 70 °C for a few minutes to increase adhesion. The PMMA on the graphene is removed using acetone and isopropyl alcohol. To fabricate the DLG/TiO_2_ and TLG/TiO_2_ nanodiodes, this process is repeated two and three times, respectively. The number of graphene layers is confirmed using the ratio of the intensity of the 2D to G peaks in the Raman spectra.

### Electrical measurement and photocurrent

For determining the electrical characteristics of the Schottky diodes, current–voltage (*I*–*V*) curves were measured by sweeping the voltage between the two electrodes. By fitting the *I*–*V* curves of the diodes to the thermionic emission equation, we obtained the Schottky barrier heights, ideality factors, and series resistances of the nanodiodes. The short-circuit photocurrent of the Schottky diodes was measured under illumination by a tungsten–halogen lamp with a normal incidence angle (9 mW/cm^2^) using a Sourcemeter (2400, Keithley Instrumentation). The active area for the photocurrent was confirmed by measuring the photocurrent at each position, including graphene/Au, graphene/SiO_2_, graphene/TiO_2_, TiO_2_, and the Au electrode. The effective photocurrent was observed on the graphene/TiO_2_ interface, as confirmed by Fig. S8.

The IPCE data were obtained on the nanodiodes using a PEC–S20. First, the IPCE of the reference Si photodiode was measured to calculate the irradiance of each wavelength. After this, the photocurrent density of the nanodiode was automatically calculated based on the standard solar spectrum (AM 1.5 G–100 mWcm^−2^) corrected by the reference Si photodiode. The IPCE of the nanodiodes was then measured based on the corrected spectra using


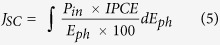


where *J*_*SC*_ is the short–circuit photocurrent density (Acm^−2^), *P*_*in*_ is the irradiance intensity at the specific photon energy, and *E*_*ph*_ is the incident photon energy.

### Doping methods

Graphene was doped with TETA and HNO_3_ for n–type and p–type properties, respectively. First, TETA was vaporized on a glass dish at 50 °C for 3 minutes on a hot plate. Finally, the TETA molecules were deposited onto the graphene surface. For HNO_3_ (63%) doping, the dipping method was used. The graphene/TiO_2_ nanodiode was dipped in HNO_3_ (63%) for 5 minutes and dried in air.

## Additional Information

**How to cite this article**: Lee, Y. K. *et al.* Hot carrier multiplication on graphene/TiO_2_ Schottky nanodiodes. *Sci. Rep.* **6**, 27549; doi: 10.1038/srep27549 (2016).

## Supplementary Material

Supplementary Information
