# Supplementary material for: Hot carrier multiplication on graphene/TiO2 Schottky nanodiodes
Source: Sci Rep. 2016 Jun 8;6:27549. doi: 10.1038/srep27549 (PMC4897609; doi:10.1038/srep27549)
Supplement: Supplementary Information [file srep27549-s1.pdf]

Supplementary information

Hot carrier multiplication on graphene/TiO_2 Schottky nanodiodes

Young Keun Lee[1,2], Hongkyw Choi[3], Hyunsoo Lee[1,2], Changhwan Lee[1,2], Jin Sik Choi[3], Choon-Gi Choi[3], Euyheon Hwang[4] and Jeong Young Park[1,2]

[1]Center for Nanomaterials and Chemical Reactions, Institute for Basic Science(IBS), Daejeon 305-701, Korea. [2]Graduate School of EEWS, Korea Advanced Institute of Science and Technology(KAIST), Daejeon 305-701, Korea. [3]Creative Research Center for Graphene Electronics, Electronics and Telecommunications Research Institute (ETRI), Gajeongno, Yuseong-gu, Daejeon, 305-700, Republic of Korea. [4]SKKU Advanced Institute of Nanotechnology, Sungkyunkwan University, Suwon 440-746, Republic of Korea. Correspondence and requests for materials should be addressed to J.Y.P. and E. H. (email: jeongypark@kaist.ac.kr, euyheon@skku.edu)

Determination of Schottky barrier heights of nanodiodes

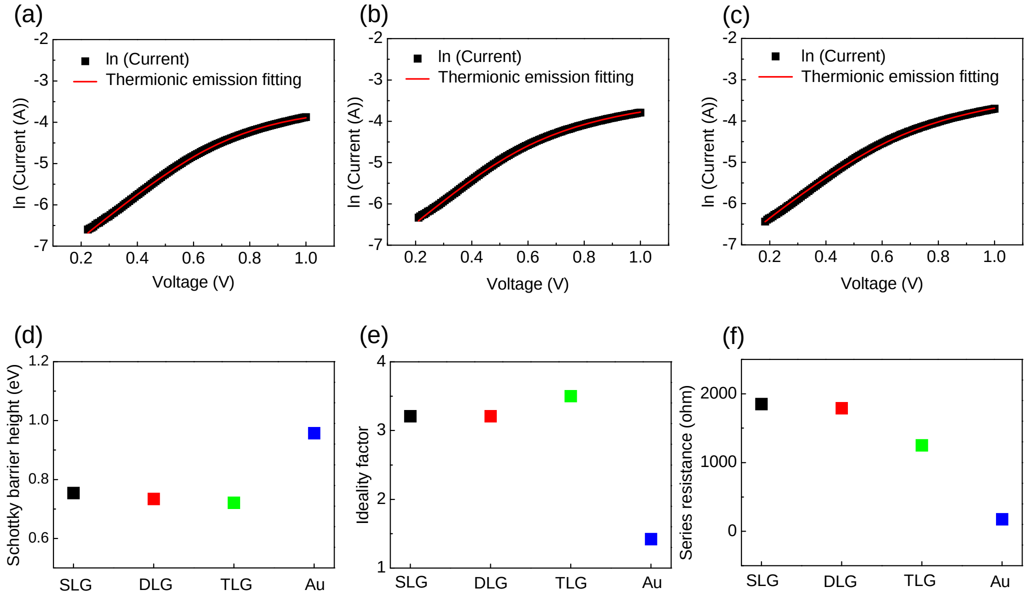

Figure S1. Thermionic emission fitting to determine the Schottky barrier heights, ideality factors, and series resistances of the nanodiodes. Plots of current versus voltage measured on (a) SLG/TiO_2, (b) DLG/TiO_2, and (c) TLG/TiO_2. (d) Schottky barrier heights, (e) ideality factors, and (f) series resistances of the graphene/TiO_2 (including Au/TiO_2) nanodiodes.

To determine the electrical factors of the nanodiodes (e.g. the Schottky barrier heights, ideality factors, and series resistances), Fig. S1 shows the electrical characteristics of the graphene/TiO_2 and Au/TiO_2 nanodiodes, which were investigated by fitting the current–voltage (*I*–*V*) curves to the thermionic emission equation. For thermionic emission over the barrier, the current through the Schottky contact as a function of applied voltage is given by

$$I = FA^{*}T^{2}\exp\left(-\frac{\Phi_{\mathrm{n}}}{k_{B}T}\right)\cdot\left[exp\left(\frac{e_{0}(V_{a}-R_{S}I)}{\eta k_{B}T}\right)-1\right]$$

where F is the area, A^{*} is the effective Richardson constant, Φ_n is the Schottky barrier height, η is the ideality factor, and R_s is the series resistance. The effective Richardson constant for

TiO_2 is 24 A/cm^2 K.[1]

Alternatively, the temperature dependence was investigated in the saturation regime to determine the Schottky barrier height of the graphene/TiO_2 and Au/TiO_2 nanodiodes. Here, the saturation current (I_{sat}) is proportional to $T^2 exp(-\frac{\Phi_n}{k_B T})$ where T is the temperature (K), k_B is the Boltzmann constant, and Φ_n is the Schottky barrier height. Figure S2 shows the *I–V* curves measured as a function of temperature. To obtain the Schottky barrier height of the nanodiode, we calculated the slope of the plot of $ln(I_{sat}/T^2)$ as a function of $e/(k_B T)$.[2,3] The Schottky barrier heights of the SLG/TiO_2 and Au/TiO_2 nanodiodes are 0.71 and 0.93 eV, respectively, as shown in Fig. S2, which are in good agreement with thermionic emission equation fitting, showing Schottky barrier heights of 0.75 and 0.98 eV, respectively.

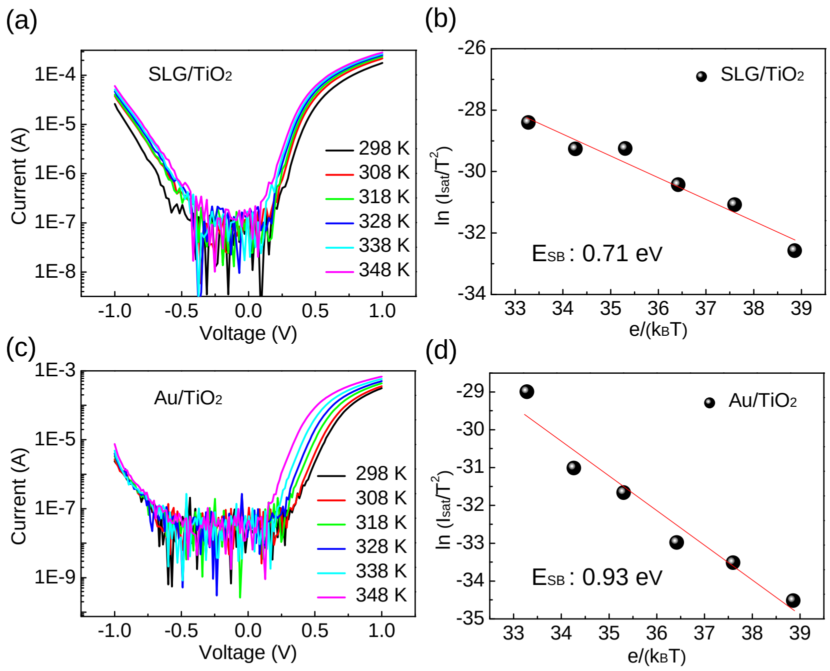

Figure S2. Determination of the Schottky barrier heights of the graphene/TiO_2 and Au/TiO_2 nanodiodes. (a) *I–V* measured on SLG/TiO_2 as a function of temperature and (b)

corresponding plot of $ln(I_{sat}/T^2)$ as a function of $e/(k_BT)$. (c) I–V measured on Au/TiO_2 as a function of temperature and (d) corresponding plot of $ln(I_{sat}/T^2)$ as a function of $e/(k_BT)$.

Raman spectra dependent on doping

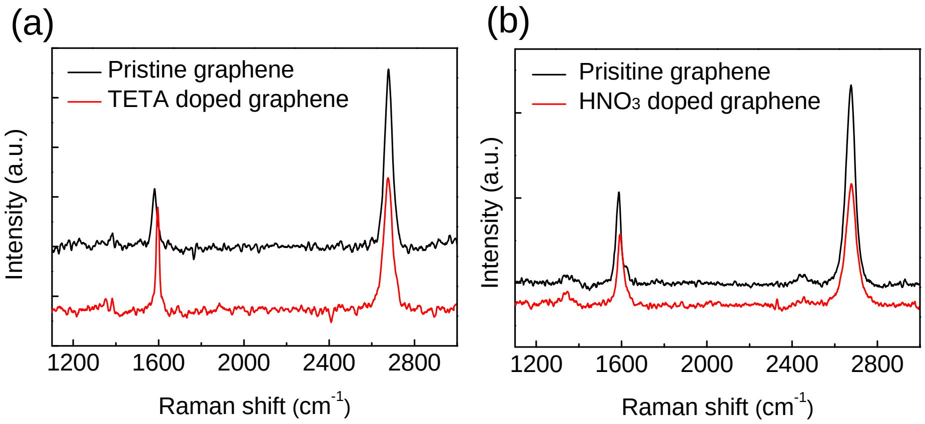

Figure S3. Comparison of Raman spectra after TETA- and HNO_3-doping of the graphene. Raman spectra measured on the (a) TETA-doped graphene and (b) HNO_3-doped graphene.

The pristine, TETA-, and HNO_3-doped graphene were investigated by Raman spectroscopy using an excitation wavelength, 514 nm laser (2.4 eV) to compare the characteristics of the Raman spectra. The pristine graphene shows two obvious Raman peaks at 1581 cm^{-1} and 2678 cm^{-1}, corresponding to the G and 2D bands, respectively, in Fig. S4a. After TETA doping, the position of the G band changed to 1596 cm^{-1}. The upshift of the G band is attributed to electron doping with amine groups, resulting in a Fermi level shift. At the same time, the position of the G band shifted from 1586 to 1595 cm^{-1} in the HNO_3-doped graphene (Fig. S4b). The intensity ratio of the 2D to G peaks decreased due to TETA and HNO_3 doping, which is additional evidence of the doping effect. In addition, after doping, the values of the FWHM decreased from 25 cm^{-1} in pristine graphene to 16 cm^{-1} in the TETA-doped graphene because of a forbidden electron–hole pair from the Pauli exclusion principle.[4] On the other hand, HNO_3 doping only shows a slight change of the FWHM of the

G band from 24 to 26 cm^{-1}.[5]

XPS spectra as a function of doping

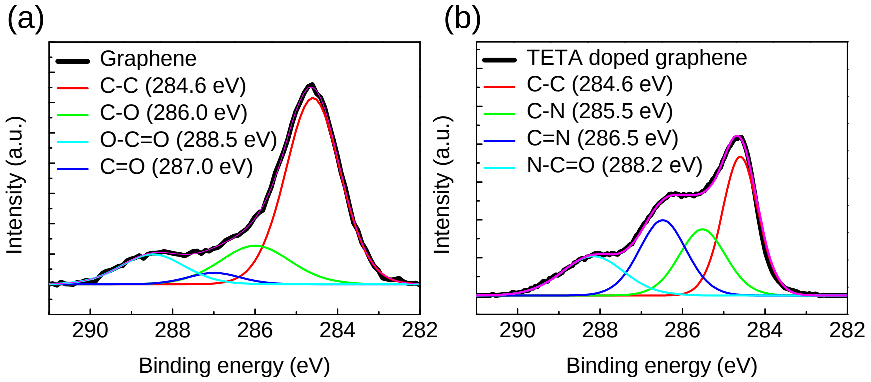

Figure S4. Comparison of XPS spectra of the C 1s of pristine and TETA-doped graphene. XPS spectra of (a) pristine and (b) TETA-doped graphene.

To confirm the doping of graphene with the amine groups of TETA, C 1s XPS spectra were obtained. In pristine graphene, the C 1s XPS spectrum exhibited noticeable peaks at 284.6 eV, 286.0 eV, 287.0 eV, and 288.5 eV corresponding to C–C, C–O, C=O, and O–C=O bonds, respectively. On the other hand, after TETA doping, a significant increase in the XPS spectrum of C 1s was observed due to bonding with the amine groups. The increase of the peaks at 285.5 eV, 286.5 eV, and 288.2 eV were respectively attributed to C–N, C=N, and N–C=O bonds, respectively, indicating TETA-induced doping.[6,7]

Thermionic emission fitting after doping

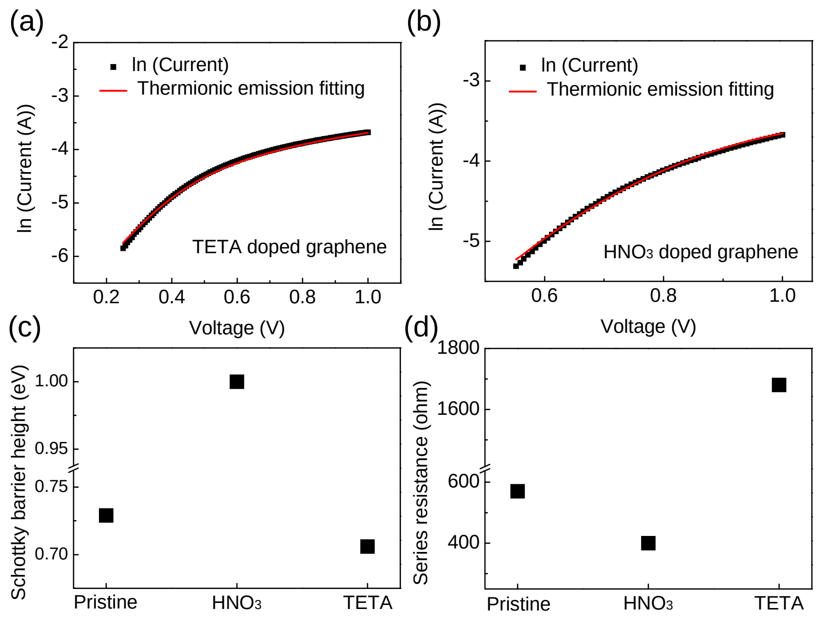

Figure S5. Thermionic emission fitting to investigate the electrical properties after (a) TETA and (b) HNO_3 doping. Changes of (c) the Schottky barrier height and (d) series resistance after TETA and HNO_3 doping.

IPCE measurements as a function of doping

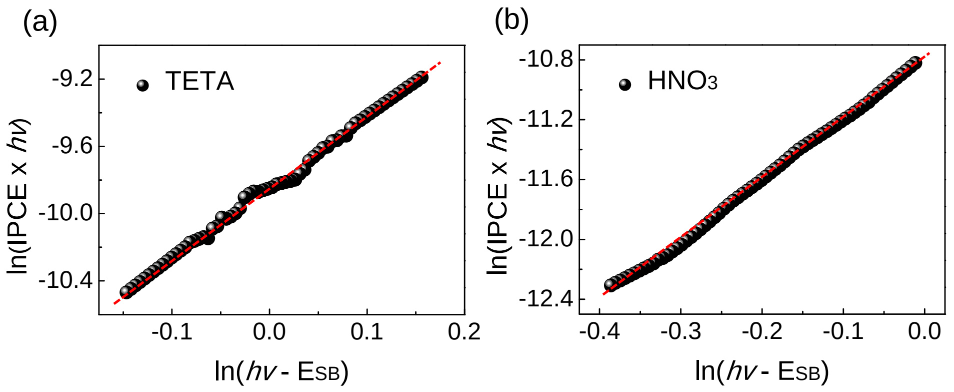

Figure S6. Plots of ln($IPCE \times hv$) as a function of ln($hv - E_{SB}$) depending on doping. (a) TETA- and (b) HNO_3-doped graphene/TiO_2 Schottky nanodiodes.

Photocurrent obtained depending on position

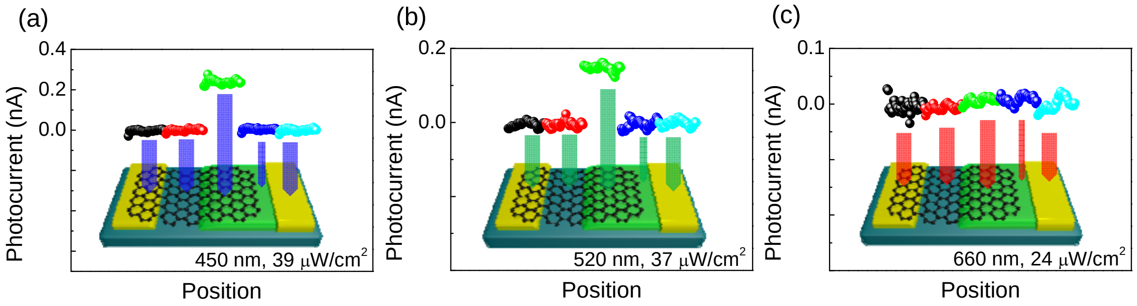

Figure S7. Photocurrent measured on the SLG/TiO_2 Schottky nanodiode as a function of position and photon energy.

The short-circuit photocurrent was measured as a function of position and photon energy. A xenon lamp was used as the light source and monochromatic light was produced by using two ruled diffraction gratings (Oriel® Tunable Light Source Systems). When the specific wavelength was incident to the normal angle on the graphene/TiO_2 Schottky diode, the short-circuit photocurrent was measured by a Sourcemeter (2400, Keithley Instrumentation). To confirm the active area where the photocurrent was generated by the photon energy, the photocurrent was measured as a function of position, such as the interfaces between the graphene/Au, graphene/SiO_2, graphene/TiO_2, the TiO_2, and the Au electrode. The effective photocurrent was obtained on the graphene/TiO_2, implying the formation of a Schottky barrier at the interface between the graphene and TiO_2 as the detection mechanism for hot electrons by carrier multiplication in the graphene.

Atomic stick–slip image of graphene

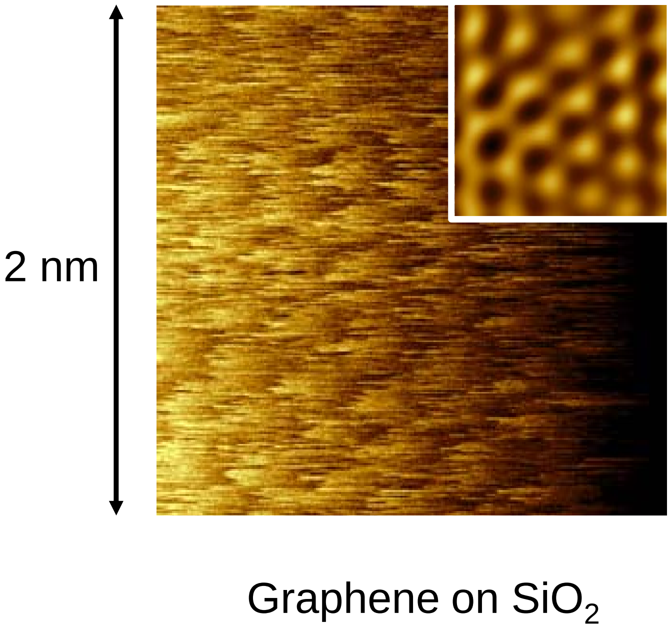

Figure S8. Fourier-filtered atomic stick–slip image of graphene on the Schottky nanodiode.

The atomic stick–slip image (2 x 2 nm^2) was obtained using friction force microscopy (Agilent 5500) at ambient conditions. A Si tip (PPP–LFMR–50) with a typical force constant of 0.2 N/m was used. The scan speed and applied normal load were 0.032 μm/s and 3.1 nN, respectively. The inset is a Fourier-filtered atomic stick–slip image.

References

1 Park, J. Y., Renzas, J. R., Hsu, B. B. & Somorjai, G. A. Interfacial and chemical properties of Pt/TiO_2, Pd/TiO_2, and Pt/GaN catalytic nanodiodes influencing hot electron flow. *J. Phys. Chem. C* **111,** 15331-15336 (2007).

2 Mtangi, W. *et al.* The dependence of barrier height on temperature for Pd Schottky contacts on ZnO. *Physica B* **404,** 4402-4405 (2009).

3 Yang, H. *et al.* Graphene barristor, a triode device with a gate-controlled Schottky barrier. *Science* **336,** 1140-1143 (2012).

4 Kim, Y. *et al.* Vapor-phase molecular doping of graphene for high-performance transparent electrodes. *Acs Nano* **8,** 868-874 (2014).

5 Das, A. *et al.* Monitoring dopants by Raman scattering in an electrochemically top-gated graphene transistor. *Nat. Nanotechnol.* **3,** 210-215, (2008).

6 Zhang, Y. Q. *et al.* One-pot synthesis of N-doped carbon dots with tunable luminescence properties. *J. Mater. Chem.* **22,** 16714-16718 (2012).

7 Tu, Q. *et al.* Effects of surface charges of graphene oxide on neuronal outgrowth and branching. *Analyst* **139,** 105-115 (2014).
